# Wait-and-see or radical surgery for rectal cancer patients with a clinical complete response after neoadjuvant chemoradiotherapy: a cohort study

**DOI:** 10.18632/oncotarget.6093

**Published:** 2015-10-12

**Authors:** Jun Li, Hao Liu, Jie Yin, Sai Liu, Junjie Hu, Feng Du, Jiatian Yuan, Bo Lv, Jun Fan, Shusheng Leng, Xin Zhang

**Affiliations:** ^1^ General Surgery Department, Affiliated Hospital/Clinical Medical College of Chengdu University, Chengdu, People's Republic of China; ^2^ 2nd Affiliated Hospital of Jilin University, Changchun, People's Republic of China; ^3^ Xuzhou Central Hospital, Xuzhou, People's Republic of China; ^4^ Beijing Youan Hospital, Capital Medical University, Beijing, People's Republic of China; ^5^ Hubei Cancer Hospital, Wuhan, People's Republic of China; ^6^ Cancer Institute/Hospital, Peking Union Medical College and Chinese Academy of Medical Sciences, People's Republic of China

**Keywords:** rectal cancer, neoadjuvant chemoradiotherapy, complete clinical response

## Abstract

A wait-and-see policy might be considered instead of surgery for rectal cancer patients with no residual tumor or involved lymph nodes on imaging or endoscopy after neoadjuvant chemoradiotherapy (clinical complete response, cCR). In this cohort study, we compared the oncologic outcomes of rectal cancer patients with a cCR who were managed according to a wait-and-see policy (observation group) or with surgery (surgery group). In the observation group, follow-up was performed every 3 months for the first year and consisted of MRI, endoscopy with biopsy, computed tomography and transrectal ultrasonography. In the surgery group, patients received radical surgery. Long-term oncologic outcomes were estimated using Kaplan-Meier curves. Thirty patients were enrolled in the observation group (median follow-up, 60 months; range, 18-100 months), and 92 patients were enrolled in the surgery group (median follow-up, 58 months; range, 18-109 months). The 5-year disease free survival and overall survival rates were similar in the two groups: 90.0% vs. 94.3% (*P* = 0.932) and 100.0% vs. 95.6% (*P* = 0.912), respectively. We conclude that for rectal cancer patients with a cCR after neoadjuvant chemoradiotherapy, a wait-and-see policy with strict selection criteria, follow-up and salvage treatments achieves outcomes at least as good as radical surgery. Additionally, we declare that the pCR (pathologic complete regression) and non-pCR subgroups of patients with a cCR have similar long-term failure (local recurrence and/or distant metastasis) rate.

## INTRODUCTION

In cases of locally advanced rectal cancer, neoadjuvant chemoradiotherapy (NCRT) can induce tumor regression [1] and reduce local recurrence [2]. Following this treatment, evidence from digital rectal examination, magnetic resonance imaging (MRI), endoscopy with biopsy and transrectal ultrasonography indicates a clinical complete response (cCR) is attained in about 26.8% of patients [2, 3, 4]. Achieving a cCR provides these patients with an opportunity to avoid radical surgery, which is associated with related complications and mortality [5]. The first prospective study of the wait-and-see policy, or observational management of rectal cancers with a cCR after NCRT, was reported by Habr-Gama et al. [6]. In that study, the clinical response to NCRT was assessed 8-10 weeks after completion of the therapy, and those with a cCR were actively observed for an additional 10 months. Patients who sustained a cCR for one year after NCRT were treated based on a wait-and-see policy. Habr-Gama et al. [6] reported that among 71 patients receiving nonoperative management, the 5-year overall survival (OS) and disease-free survival (DFS) rates were 100% and 92%, respectively, and the local recurrence (LR) rate was 3%. Although this was a small study, the wait-and-see policy attracted much interest among clinicians, and Mass et al. [7] confirmed the efficacy of a nonoperative approach using MRI and endoscopy with biopsy to evaluate clinical responses. The purpose of our study was to evaluate and compare the oncologic outcomes of rectal cancer patients who, after treatment with NCRT, achieved a cCR and were then managed according to a wait-and-see policy or treated surgically.

## RESULTS

### Characteristics of patients in the surgery and observation groups

In total, 122 patients showed a cCR 8-10 weeks after completing NCRT and were enrolled in the study. The male/female ratio was 18/12 in the observation group and 60/32 in the surgery group. The median ages of the two groups were 62.0±4.3 years (range: 55-82) and 56.0±9.2 (range: 34-73), respectively. The clinical characteristics of the observation and surgery groups are listed in Table 1. Patients in the two groups were similar with respect to age, gender, morbidity (including chronic hepatitis and diabetes mellitus), distance of tumor from the anal verge, serum CEA levels before NCRT, cT stage and cN stage (*P* > 0.05 in all cases).

**Table 1:** Characteristics of patients with a cCR after NCRT

Variable	Observation Group		Surgery Group		All Cases		χ^2^	P
	No. of Cases	%	No. of Cases	%	No. of Cases	%		
Patients (n=122)	30	24.6	92	75.4	122	100.0		
Age (median, range, year)	62 (55-82)		56 (34-73)		58 (34-82)		3.405*	0.076
Gender								
Male	18	23.1	60	76.9	78	63.9	0.267	0.605
Female	12	27.3	32	72.7	44	36.1		
Morbidity								
Yes	10	28.6	25	71.4	35	28.7	0.420	0.517
No	20	23.0	67	77.0	87	71.3		
Distance from anal verge (mean, range, cm)	3.5 (0-7)		3.8 (0-7)		3.7 (0-7)		0.203*	0.652
Serum CEA levels								
Positive	8	25.8	23	74.2	31	25.4	0.033	0.856
Negative	22	24.2	69	75.8	91	74.6		
cT stage								
T1	3	23.1	10	76.9	13	10.7	0.093	0.993
T2	5	26.3	14	73.7	19	15.6		
T3	15	23.8	48	76.2	63	51.6		
T4	7	25.9	20	74.1	27	22.1		
cN stage								
N0	14	26.4	39	73.6	53	43.4	0.168	0.682
N+	16	23.2	53	76.8	69	56.6		

Abbreviations: Age and Distance from anal verge tested using t-test,

Morbidity includes chronic hepatitis and diabetes mellitus.

### Observation group

The observation group consisted of 30 patients with advanced rectal cancer who had a cCR after completing NCRT and were then treated with nonoperation management. The initial mean distance of their tumors from the anal verge was 3.5 cm (0-7 cm). At clinical and radiological staging prior to treatment, 3 (10.0%) patients had T1 lesions, 5 (16.7%) had T2 lesions, 15 (50.0%) had T3 lesions, and 7 (23.3%) had T4 lesions. Sixteen (53.3%) patients had radiological evidence of N+ lesions (Table 1).

The mean follow-up period in the observation group was 58 months (19-108). All 30 patients had at least 12 months of follow-up, and 93.3% of patients (28/30) had at least 24 months of follow-up. The numbers of patients followed-up at yearly intervals are listed in Table 2. Two (6.7%) patients developed LR 18 and 26 months after completing NCRT. The first was treated with salvage dissection (TME) and was alive without LR or distant metastasis (DM) 37 months after the surgery. The second was managed with local excision and was alive without LR or DM after 62 months of follow up. One patient (3.3%) developed DM after 50 months of follow-up, and was alive after 67 months. No patient experienced both LR and DM. The 5-year DFS and OS rates were 90.0% and 100.0%, respectively (Table 5).

**Table 2:** Follow-up at yearly intervals

Follow-up(month)	Observation Group		Surgery Group	
	No. of Cases	%	No. of Cases	%
12	30	100.0	92	100.0
24	28	93.3	85	92.4
36	24	80.0	72	78.2
48	20	66.7	61	66.3
60	15	50.0	45	48.9
72	8	26.7	33	35.9
96	2	6.6	8	8.7
108	1	3.3	1	1.1

### Surgery group

Ninety-two patients with a cCR after completing NCRT were enrolled into the surgery group. The initial mean distance of their tumors from the anal verge was 3.8 cm (0-7 cm) (Table 1). The mean follow-up period was 58 months (18-108), and 92.4% (85/92) of patients had a minimum of 24 months of follow-up (Table 2). At pretreatment clinical and radiological staging, 10 (10.8%) patients had T1 lesions, 14 (15.2%) had T2 lesions, 48 (52.7%) had T3 lesions, and 20 (21.7%) had T4 lesions. Fifty-three (57.6%) patients had radiologic evidence of N+ lesions (Table 1). Eighty-one patients (88.0%) showed pathologic complete regression (pCR, no residual tumor cells in the specimens) after full pathologic examination of the specimens, while the other 11 patients did not (Table 3). According to Dworak's TRG system, 5 of the 11 non-pCR patients showed TRG 0 with LN+, and 6 showed TRG1 without positive lymph nodes (not shown in the list). The pCR and non-pCR subgroups had similar 5-year failure (LR and/or DM) rates (*P* = 0.350) (Table 4).

**Table 3:** Association of 5-year failure with pCR and non-pCR in the surgery group

pCR or Not	No. of Patients		5-year Failure		χ^2^	*P*
	No. of Cases	%	No. of Cases	%		
pCR	81	88.0	6	7.4	0.872	0.350
non-pCR	11	12.0	1	9.1		
Sum	92	100.0	7	7.6		

**Table 4:** Operations performed in the surgery group

Surgery Performed	Patients		
	No. of Cases	%	
APR	40	43.5	67.3% fistula (62)
LAR with enterostomy	22	23.9	
LAR without enterostomy	30	32.6	
Total	92	100.0	

Forty (43.5%) of the patients in the surgery group were treated with APR and the remaining 52 (56.5%) were treated with sphincter-saving surgery. Among the latter, 22 (23.9%) received LAR with enterostomy. Sixty-two (67.3%) patients developed a fistula, either temporary or definitive (Table 4). No patient experienced perioperative mortality or significant surgery-related morbidity requiring reoperation. Two (2.2%) patients developed local recurrence at 24 and 30 months, respectively. The first was treated with APR and was alive for 48 months after NCRT. The second received local excision and was alive without further LR or DM after 55 months of follow up. Five (5.4%) patients developed DM within 5 years, and 1 (1.1%) patient developed DM after 67 months of follow-up. Four patients showed DM after 35, 40, 50 and 55 months of follow-up, and died after 48, 50, 59 and 67 months, respectively, from diseases related to rectal cancer. The all failure and cancer-related mortality rates were 8.7% (8/92) and 4.3% (4/92), respectively. No patients developed both LR and DM. The 5-year DFS and OS rates were 94.3% (7/92) and 95.6% (88/92), respectively (Table 5).

**Table 5:** Five-year outcomes among patients treated with wait-and-see policy or radical surgery

Groups	No. of Patients	5-year Local Recurrence		*P*	5-year Distant Metastasis		*P*	5-year Disease Free Survival		*P*	5-year Overall Survival		*P*
		No. of Cases	%		No. of Cases	%		No. of Cases	%		No. of Cases	%	
Observation group	30 (24.5%)	2	6.7	0.267	1	3.3	0.658	27	90.0	0.932	30	100.0	0.912
Surgery group	92 (75.5%)	2	2.2		5	5.4		85	94.3		88	95.6	
Sum	122 (100.0%)	4	3.3		6	4.9		112	91.8		119	97.5	

### Prognoses in the observation and surgery groups

**Figure 1:**
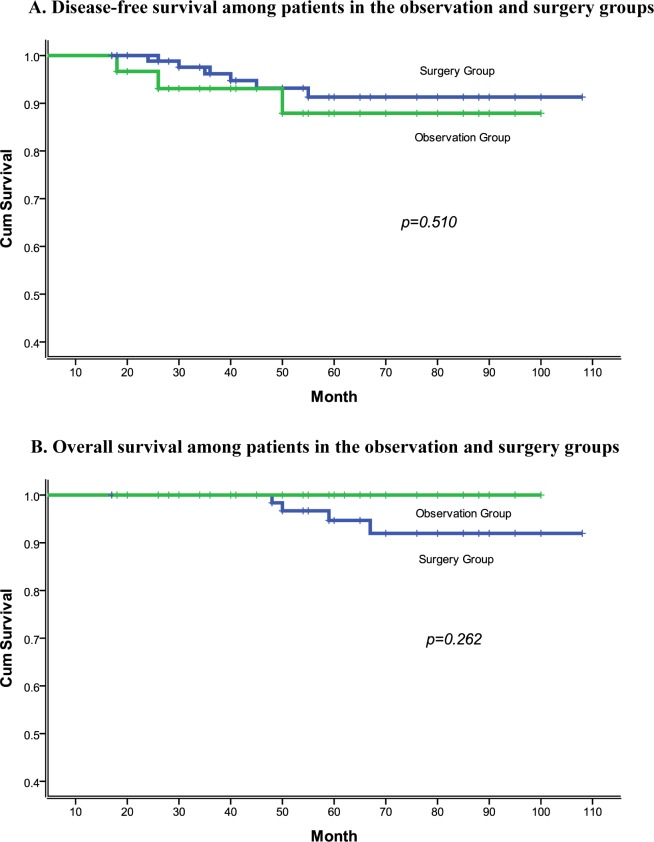
**A.** Disease-free survival among patients in the observation and surgery groups. **B.** Overall survival among patients in the observation and surgery groups.

The LR, DM, all failure and 5-year DFS rates did not differ between statistically between the observation and surgery group (*P* > 0.05). Although there were no cancer-related deaths in the observation group, Kaplan-Meier analysis showed there to be no significant difference in 5-year OS between the surgery and observation groups (*P* = 0.262) (Figure 1A, 1B).

## DISCUSSION

The wait-and-see policy for rectal cancer patients with a cCR after NCRT is based on careful selection and follow-up using endoscopy and up-to-date imaging, and appears both feasible and safe. The pursuit of this approach was inspired by the pioneering work of Habr-Gama et al. [8] and a second study reported by Mass et al. [7]. From their data, it is apparent that NCRT can result in tumor downstaging and may lead to cCR, or even pCR. A pCR is achieved in 15%-40% of patients treated with NCRT [9,10]. Moreover, the development of improved NCRT protocols has increased the number of patients achieving a cCR [4], and our study provides meaningful insight into the management of rectal cancer patients with a cCR.

In this series, only two patients developed LR. Both patients were treated with salvage therapy and both remained alive without further LR or DM throughout the follow-up period. The oncologic prognoses of patients with a cCR and managed according to the wait-and-see policy were comparable to those of patients with a cCR after radical surgery. However, the functional outcomes were significantly better for patients treated using the observation strategy. Thus our experience is strongly indicative of the benefit of a wait-and-see approach with salvage surgery when necessary, as well as the need for careful monitoring of the rectum.

One of the major concerns regarding the wait-and-see policy is how tumor regression after NCRT can be assessed more efficiently by clinicians. Dalton et al. [11] reported on 6 patients in whom tumor recurrence was detected through clinical examination and MRI after a median follow-up of 28 months. This is the first published data to show that imaging may improve assessment of patients with a cCR. In our study, cCR was determined almost entirely based on digital rectal examination, CT, MRI, endoscopy with biopsy and transrectal ultrasonography. Among the surgery group, 81 members proved to have a pCR, while residual tumors were detected in surgical specimens from 11 patients. The accuracy of the assessment of cCR was thus 88.0% (81/92).

Another issue is whether a cCR can accurately predict pCR. From published data and expert opinion, including our study, it is clear that cCR may not mean pCR. Moreover, following NCRT, up to 7% of patients may have a pCR, despite an incomplete clinical response characterized by a residual rectal ulcer [12]. The reason for this discrepancy remains unknown. According to Dworak's TRG system, we found that 5 of the 11 non-pCR patients showed TRG 0 with LN+, while 6 showed TRG1 without positive lymph nodes. However, the pCR and non-pCR subgroups had similar 5-year failure (LR and/or DM) rates (*P* = 0.350). This indicates that the prognosis of patients with a cCR after careful examination using MRI, endoscopy, and transrectal ultrasonography is as good as that for patients with a pCR. But still, a more accurate and efficacious method for assessing whether residual tumor cells persist is needed.

As in earlier studies, all patients in the surgery group (control group) showed a pCR after surgery. There are solid evidences that pCR is predictive of a good prognosis. In the present study, however, all of the patients in the observation group had only a cCR, and there is not yet enough evidence to predict clinical outcome based on a cCR. One major advantage of this study is that at the time of recruitment, all the patients in both groups had a cCR. This is unlike earlier studies, in which patients in the surgery group had a pCR, while those in the wait-and-see group had only a cCR. That feature of the earlier studies could lead to bias, as the patients in the two groups had different start points. The factors contributing to the outcome similarity observed in our study may include improvements in endoscopic and imaging techniques for tumor detection that improve the ability of NCRT to eliminate involved lymph nodes, improvements in peri-radiotherapy medication, improved health care strategies used both by clinicians and during home care, and improvements in diet.

The results from this study are constrained by all the flaws and biases inherent to a nonrandomized trial. In addition, the limited number of patients enrolled in this study increases the potential for bias. Nevertheless, we believe our outcome data for the wait-and-see policy are encouraging and justify prospective evaluation in larger studies. The ideal trial design to assess the efficacy and safety of the wait-and-see policy would be a randomized clinical trial comparing rectal resection with the principles of TME. Such a trial may be challenging to perform, as patients who receive a wait-and-see policy will need to be carefully selected by clinicians in collaboration with specialists in advanced imaging.

In sum, the selective use of the wait-and-see policy for rectal cancer patients achieving a cCR after NCRT appears to produce oncologic outcomes similar to those obtained when patients with a cCR receive radical surgery, while avoiding fistulas and other morbidities of surgery. Additionally, we conclude that the pCR and non-pCR subgroups of patients with a cCR before surgery have similar long-term failure rate, but the conclusion needs more effective data to validate. A more accurate and efficacious method for assessing whether residual tumor cells persist and further prospective studies are now needed to fully evaluate this promising treatment option.

## PATIENTS AND METHODS

Approval was obtained from the appropriate ethics committees at all participating study centers before the study was started. Nine hundred patients with resectable (stage II and III) distal rectal adenocarcinoma (0-7 cm from the anal verge) received NCRT (50 Gy/25 f/2 Gy, capecitabine, 825 mg/m^2^ bid, concurrently) at five study centers between April 2006 and October 2013. Pretreatment staging was based on digital rectal examination, chest radiography, abdominal and pelvic computed tomography, endoscopy, transrectal ultrasound and MRI. Eight to ten weeks after completing the NCRT, the primary tumor was reassessed using endoscopy with biopsy, MRI and transrectal ultrasound. In particular, endoscopic examination of the entire large bowel was performed in patients who first presented with obstructive tumors. An initial cCR was determined based on 1) the absence of a palpable lesion on digital rectal examination, 2) endoscopy showing no visible lesion other than a flat scar, and 3) the absence of evidence of a residual tumor on pelvic computed tomography, transrectal ultrasound or MRI. Ultimately, 13.6% (122/900) of patients were deemed to have a cCR and were recruited for this study.

### Wait-and see-policy

Eight (26.7%) patients with a cCR received non-operative treatment because of religious reasons, fistula or poor physical condition, while twenty two (73.3%) patients were managed with a wait-and see-policy on the suggestion from clinicians because of the status of no any evidences of LR and/or DM. Patients in the surgery group prefer to receive surgery instead of the “wait- and- see” policy. The observation group was referred to monthly follow-up visits for digital rectal examination and measurement of serum CEA levels. Endoscopy with biopsy (as far as possible) and transrectal ultrasonography were performed every 3 months. In addition, abdominal and pelvic CT, MRI and chest radiography were repeated every 6 months beginning 1 year after completing NCRT. Patients in the observation group who sustained cCR for at least 1 year were referred to follow-up visits every 6 months during the second and third years after completing NCRT. After 3 years, follow-up visits were yearly. If any evidence of recurrence and/or metastasis was detected, salvage treatments, including radical surgery, local excision, radiotherapy with or without chemotherapy, or chemotherapy alone were carried out according to the situation of the patients.

### Pathologic examination

Sections from all resected specimens were examined by local pathologists from five hospitals. The standardized protocol included determination of the AJCC TNM classification, stage grouping, number of examined and involved lymph nodes, presence or absence of lymphatic or venous invasion, tumor deposits and tumor regression grade (TRG). The reference pathologist tested pathological sections and then recorded the findings in a standardized document.

### Follow-up

An intensive follow-up protocol consisting of digital rectal examination, MRI, CT, endoscopy (with biopsy), and measurement of serum CEA levels was incorporated into the wait-and-see policy. The follow-up results are provided in Table 2. The follow-up end date was March 2015. The median duration of follow-up was 59 months (18-108 months).

### Statistical analysis

Information on baseline characteristics was collected and compared using χ^2^ and t tests. LR and DM were analyzed in all eligible patients in the two groups. All time-to-event end points were measured from the end of NRCT. DFS was calculated from radical resection to the discovery of any evidence of LR and/or DM. Statistical analysis was performed using SPSS software (version 18). Differences were evaluated using the log-rank test. LR and DM were analyzed as cumulative incidences. Two-sided *P* values less than 0.05 were considered significant.
